# The Healing Effect of Licorice on *Pseudomonas aeruginosa* Infected Burn Wounds in Experimental Rat Model

**Published:** 2014-07

**Authors:** Nader Tanideh, Pedram Rokhsari, Davood Mehrabani, Soleiman Mohammadi Samani, Fatemeh Sabet Sarvestani, Mohammad Javad Ashraf, Omid Koohi Hosseinabadi, Shahram Shamsian, Nasrollah Ahmadi

**Affiliations:** 1 Shiraz Burn Research Center, Pharmacology Department, Shiraz University of Medical Sciences, Shiraz, Iran;; 2 Student Research Committee, School of Medicine, Shiraz University of Medical Sciences, Shiraz, Iran;; 3 Stem Cell and Transgenic Technology Research Center, Shiraz University of Medical Sciences, Shiraz, Iran;; 4 Nano Drug Delivery Research Center, Faculty of Pharmacy, Shiraz University of Medical Sciences, Shiraz, Iran;; 5 School of Veterinary Medicine, Shiraz University, Shiraz, Iran;; 6 Pathology Department, Shiraz University of Medical Sciences, Shiraz, Iran;; 7 Laboratory Animal Center, Shiraz University of Medical Sciences, Shiraz, Iran;; 8 Mad Veterinary Clinic, Shiraz, Iran

**Keywords:** Burn, Wound, Healing, Licorice, Silver sulfadiazine, *P. aeruginosa*

## Abstract

**BACKGROUND:**

Burn is still one of the most devastating injuries in emergency medicine while improvements in wound healing knowledge and technology have resulted into development of new dressings. This study was undertaken to evaluate the healing effect of licorice in *Pseudomonas aeruginosa* infected burn wounds of experimental rat model.

**METHODS:**

One hundred and twenty female Sprague-Dawley rats were randomly allocated to 4 equal groups. Group A received silver sulfadiazine ointment, Group B received 10% licorice extract and Group C was considered as control group and received gel base as the base of medication. Group D did not receive any medication and just underwent burn injury. A standard 3rd degree burn wound was produced by a hot plate with similar size about 20% of total body surface area (TBSA) and at identical temperature. After 24 h of burn production, 10^8^ colony forming units (CFU) of toxigenic strains of *P. aeruginosa *(PA 103) were inoculated subcutaneously into the burnt area. After 3, 7, 14, 21 and 28 days of therapy, the animals were sacrificed and burn areas were macroscopically examined and histologically evaluated.

**RESULTS:**

Decrease in size of the burn wounds, in inflammation and re-epithelialization were poor in groups B-D. Infection to *P. aeruginosa* was still visible in groups B-D but was absent in Group A. The mean histological score, tensile strength, maximum stress, yield strength and stiffness in groups B-D were lower compared with Group A.

**CONCLUSION:**

Licorice extract in 10% concentration was shown not to be effective in healing of *P. aeruginosa *infected burn wounds.

## INTRODUCTION

Burn injury as a major cause of death and disability has a high cost in health care and during pregnancy can increase the mortality and morbidity more in both mother and infant.^1^^,^^2^ Healing in burn wounds is still a challenge though many medications have been introduced in the literature. So every effort is made to provide a shorter in-patient care for the burn patients.^3^

*Pseudomonas aeruginosa* as an important cause of nosocomial infection may lead to septicemia and death in burn patients emphasizing its public health importance.^4^ Topical anti-bacterial agents and disinfectants were shown to be protective against infection, but the occurrence of allergic reactions and skin irritations to these agents have reduced the rate of skin regeneration and resulted into an increase in the recovery time.^5^^,^^6^ Silver sulfadiazine (SSD) was shown as the most widely used topical treatment in burn injury with anti-microbial effects.^7^^-^^10^ A delayed wound healing following treatment may be the most important clinical adverse effect of SSD. Also, renal toxicity, leukopenia and increase in patient care cost may be noticed after administration of SSD, so SSD is not recommended to be used for long periods of time.^11^ Therefore, there is a need for alternative burn dressings and more effective therapeutic drugs for burn patients.^12^

Herbal medicine products seem to have therapeutic efficacy and to be with less toxicity and are less expensive when compared with synthetic drugs. There are several reports on using herbal drugs in healing of burn injuries.^10^^,^^13^^-^^20^

Licorice (*Glycyrrhiza glabra* L.; Family: Papilionaceae/Fabaceae) is a traditional medicinal sweet and soothing herb growing in several parts of the world. It has a large number of ingredients including ﬂavonoids, saponins, triterpene, isoﬂavonoids and chalcones, with glycyrrhizic acid being its main biologically active component.^21^ β-glycyhrritinic acid was reported as the major metabolite of glycyrrhizin.^22^ It was found that glycyrrhizic and aglycone glycyrrhetinic acids present in the licorice root to be responsible for many biological activities of this herbal medicine.^23^^-^^26^

It was shown that licorice has anti-inflammatory, anti-bacterial, antioxidant, anti-arrhythmic, anti-viral and expectorant properties^23^^-^^28^ and to be effective in detoxification and protection of the liver.^29^ This medicinal herb has been used in treatment of respiratory problems including coughs, hoarseness, sore throat and bronchitis.^30^^,^^31^ Due to lack of enough findings in the literature on healing effect of licorice in burn wounds, his study was undertaken to determine the healing effect of in *P. aeruginosa* infected burn wounds of experimental rat model.

## MATERIALS AND METHODS

One hundred and twenty male Sprague Dawley rats (250±50 g), aged between 8 and 10 weeks from Laboratory Animal Center of Shiraz University of Medical Sciences, Shiraz, Iran were enrolled. The animals were maintained under controlled environment at 22±2°C, with 55±5% of humidity and 12-h light-dark cycle. Standard laboratory chow and tap water were available ad libitum. All experiments, animal selection, subsequent care and the sacrifice procedure were identical and adhered to the guidelines of Animal Care Committee of Iran Veterinary Organization. Experiments were all performed under aseptic conditions and the protocol of anesthesia, surgical procedures, and postoperative cares were similar for all animals and during the experiments, the animals were housed one per cage. 

The root of Licorice was collected from Shiraz in the Fars province of Iran. The root of licorice were dried at room temperature and powdered in a grinder. Aqueous ethanol (75%) was added to the powdered materials (500 g), and stirred for one hour. The mixture was kept at room temperature for 48 hours. Following filtration, ethanol was evaporated under reduced pressure at 40ºC. The remained water extract was dried at oven temperature of 50ºC. Finally, 10% licorice hydroalcoholic extract was prepared in pharmacy laboratory. 

Rats were anaesthetized with ketamine (100 mg/kg) and xylazin (10 mg/kg) and the hair on the back was shaved. After sedation, the back hair of animals was shaved and the skin was cleansed with povidone iodine solution and wiped with double distilled water. A standard 3rd degree burn wound was produced by a hot plate with similar size about 20% of total body surface area (TBSA) and at identical temperature as described by Manafi et al. (2009).^4^

After 24 h of burn production, 10^8^ colony forming units (CFU) of toxigenic strains of *P. aeruginosa *(PA 103) were inoculated subcutaneously into the burnt area as described by Hazrati et al. (2010).^14^ The animals in all groups were supervised in their cages for 28 days. A sample was provided from the infected areas using sterile swabs and saline and was checked for the presence of *P. aeruginosa* after 3 days.

One hundred and twenty female Sprague-Dawley rats were randomly allocated to 4 equal groups. Group A received silver sulfadiazine (SSD) ointment, Group B received 10% licorice extract and Group C was considered as control group and received gel base as the base of medication. Group D did not receive any medication and just underwent burn injury. Tissue samples were provided after 3, 7, 14, 21 and 28 days of therapy for further histological evaluation. 

The animals were sacrificed with an overdose of anesthetics and the burn areas were removed and transferred in 10% neutral-buffered formalin until tissue processing for histological evaluation as described by Abramov et al. (2007) (Table 1).^32^ The specimens were embedded in paraffin, and sections of 5 µm in thickness were provided and stained using hematoxylin and eosin (H&E) and studied by a routine light microscope (Olympus, Tokyo, Japan). 

Skin tensile strength was determined as described by Jimenez and Rampy (1999) and Rashed et al. (2003)^33^^,^^34^ for the amount and quality of synthesized collagen, as well as degradation of preformed collagen. Tensile strength demonstrated the needed force per unit of cross-sectional area to break the wound, reflecting the subdermal organization of the collagen fibers in the newly deposited collagen.

**Table 1:** Histological scoring ofwound healing

**Indices**	**Scores**				
	**1**	**2**	**3**	**4**	**5**
Epithelialization	None	None	Partial	Complete, immature	Complete, mature
Collagenization	None	None	Partial	Complete, irregular	Complete, regular
Inflammation	Severe	Moderate	Mild	None	None
Neovascularization	None	None	<5/HPF	6-10/HFP	>10/HFP
Necrosis	Extensive	Focal	None	None	None
Granulation tissue	None	Immature	Mild mature	Moderate mature	Full mature

The skin samples were obtained immediately after euthanasia and placed between sterile sponges soaked with 0.9% saline to preserve the normal tissue hydration after sampling. They were then placed in occlusive bags and stored at –70°C prior to evaluation. Biomechanical testing was performed as described by Trudel et al. (2009)^35^ by Tensile Testing Machine (Hounsfield, England). Each sample was loaded by elongating it at a displacement rate of 20 mms−1. The ultimate tensile strength, yield strength, stress and stiffness were measured. The stress value (N mm–2) was calculated by dividing the ultimate strength (N) by the cross-sectional area (mm–2). Stiffness (Nmm−1) was calculated by fitting a linear regression line to the load–deformation data from 30% to 90% of the ultimate tensile strength on the deformation curve.

Data are expressed as mean±SEM, and statistical significance between experimental and control values was analyzed by Mann-Whitney U test, One-way analysis of variance (ANOVA) and Tukey post test using SPSS software (Version 16, Chicago, IL, USA). A P value <0.05 was considered statistically significant.

## RESULTS

**Fig. 1:**
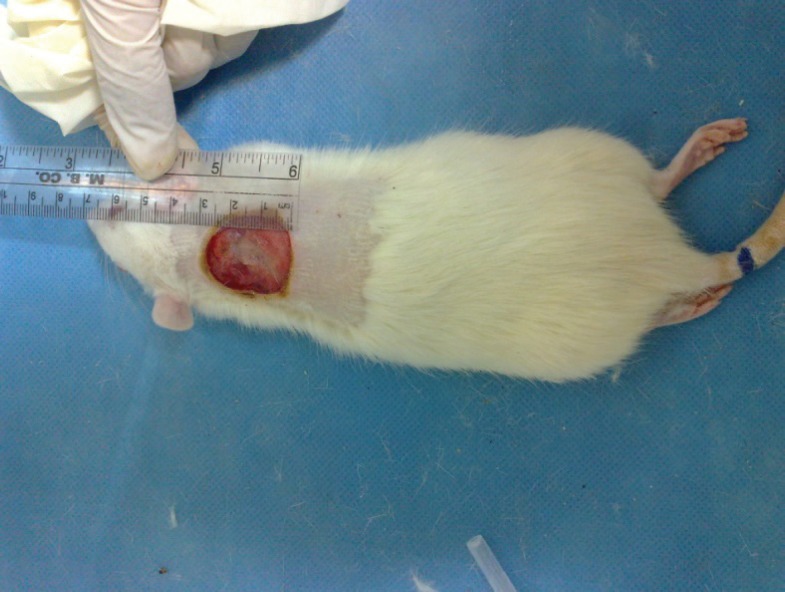
One day after burn injury in Group 4.

**Fig. 2:**
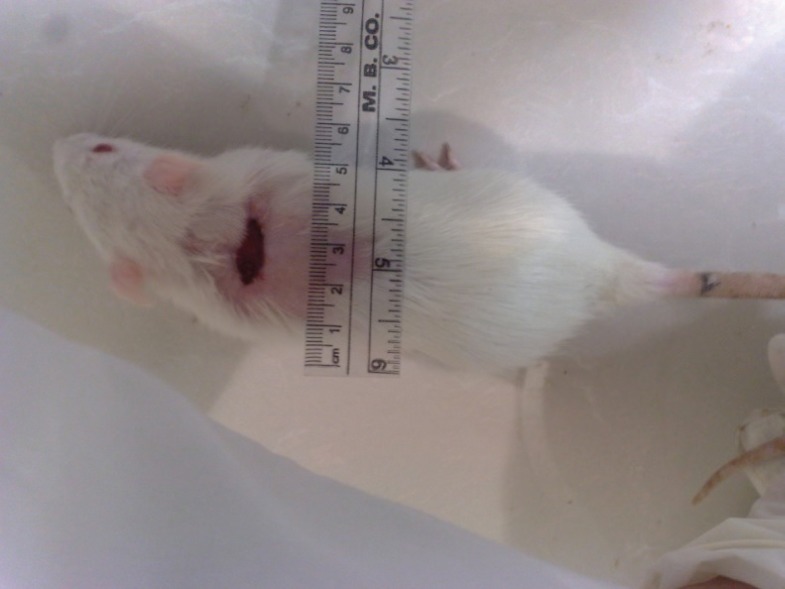
Fourteen days after burn injury in Group B showing lack of enough wound contraction.

**Fig. 3:**
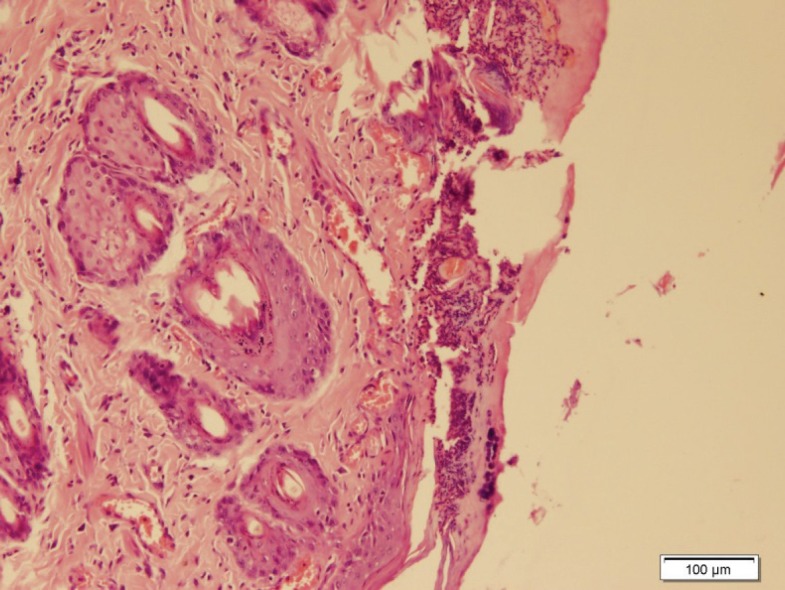
Fourteen days after burn injury in Group D (H&E, ×100).

**Fig. 4:**
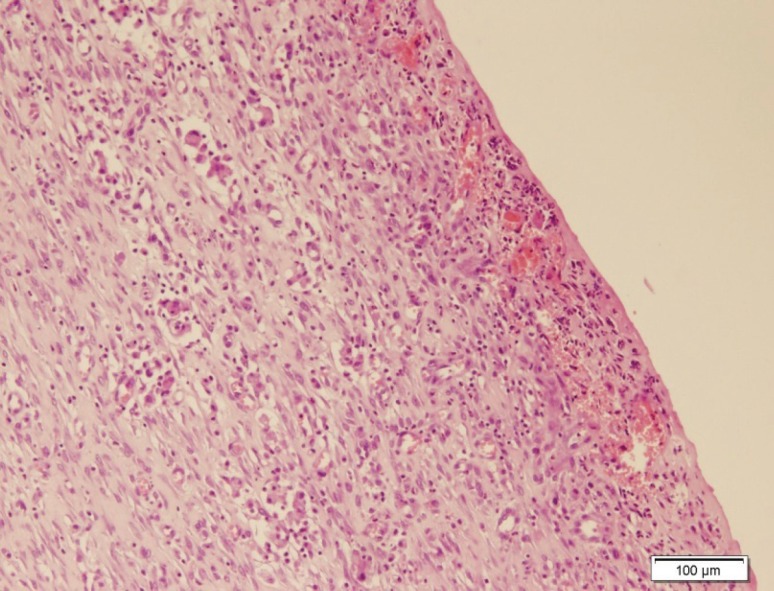
Fourteen days after burn injury in Group A (H&E, ×100).

**Fig. 5:**
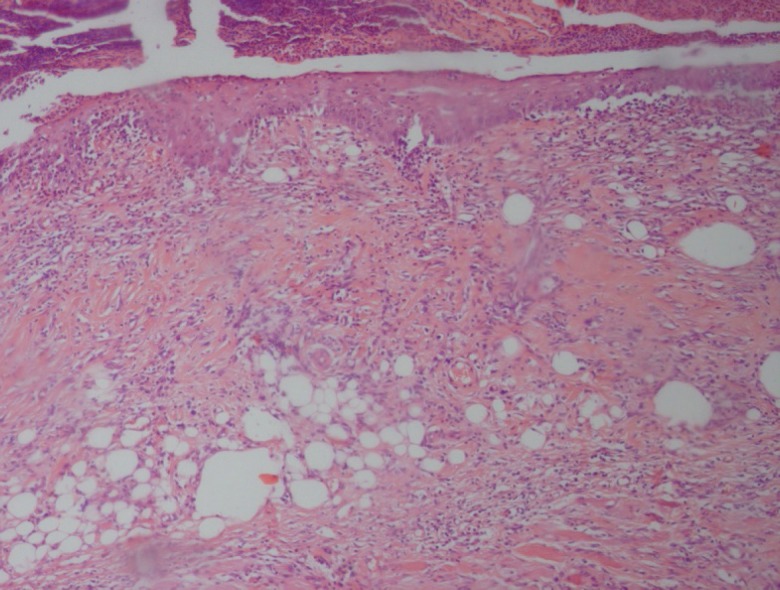
Fourteen days after burn injury in Group B with poor granulation tissue formation, partial epithelialization and irregular collagen fibers (H&E, ×100).

**Fig. 6:**
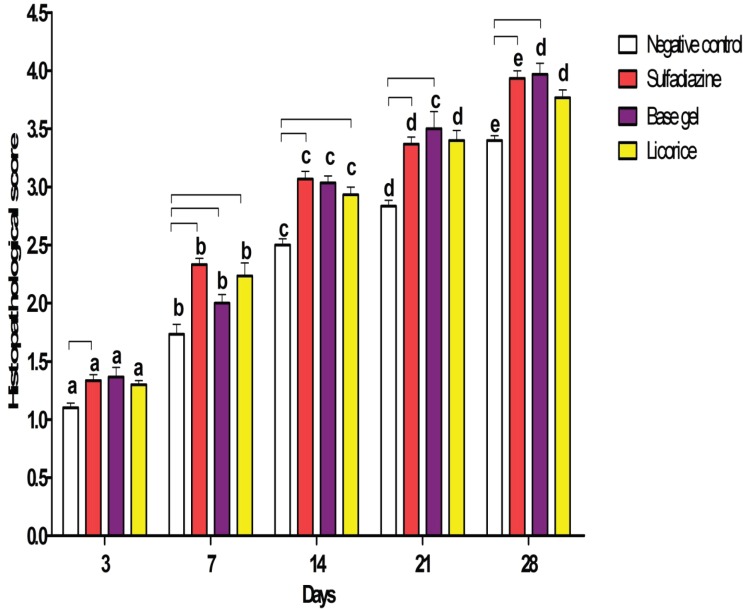
Histopathological scoring (mean±SEM) comparing licorice with other groups after treatment of burn injury in different intervals.

Burn injury in Group D (control group) was macroscopically shown in Figure 1 while in Group B after 14 days post-burn injury, there was still a lack of enough wound contraction as a process of shrinkage of the area of the wound (Figure 2). Microscopically, 14 days after burn injury in Group D, coagulative necrosis of epidermis with dilatation of capillaries in papillary dermis were noted. No reepithelialization and granulation tissue formation were identified (Figure 3). Fourteen days after treatment with SSD (Group A), presence of granulation tissue with severe acute and chronic inflammation were noted. No evidence of reepithelialization was identified. (Figure 4) while in Group B after administration of licorice, there were nearly complete reepithelialization , minimal granulation tissue formation and mild acute and chronic inflammation. Irregular collagen deposition was also identified (Figure 5 and 6). No infection was visible in group treated with SSD, but in other groups, infection to *P. aeruginosa *was visible. 

**Fig. 7:**
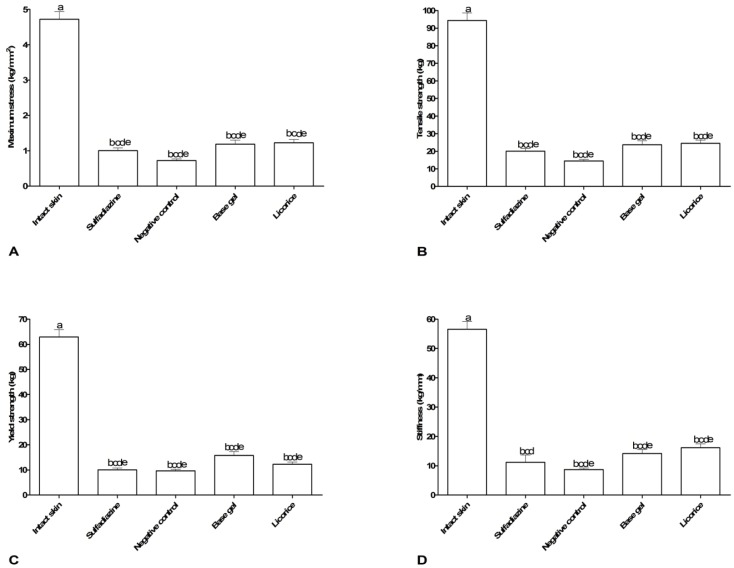
Biomechanical performance (mean±SEM) comparing licorice with other groups after 28 days of burn injury treatment

The biomechanical findings were presented in Figure 7. Treatment with licorice extract could not increase the tensile strength, maximum stress, yield strength and stiffness in comparison to other groups after 28 days.

## DISCUSSION

Burn wound healing is a process by which the damaged tissue is restored as closely as possible to its normal state with a wound contraction as a process of shrinkage of the area of the wound being dependent upon the ability of the tissue to repair, the type and extent of damage and the general state of health.^36^ Heat denaturation, such as in thermal burn, destroys the helical structure of the collagen molecule and if the temperature is sufficiently high, cleaves the Schiff-base bond thus solubilizing a fraction of intact collagen as a high molecular weight gelatin.^37^

SSD was proved to be an effective anti-bacterial agent but was shown to delay the healing process in burn wounds due to inhibition of proliferation of the keratinocytes and fibroblasts leading to impairment of the wound-healing process.^38^ We found identical finding in healing of third degree burns infected to *P. aeruginosa* after application of SSD. 

It was shown that 1% and 2% licorice topical gel were effective in reduction of erythema, edema and itching after two weeks.^39^ Several studies revealed anti-inﬂammatory,^40^^,^^41^ and anti-tumor^42^ effects of licorice that may be due to steroid structure of glycyrrhizin and α -and β -glycyrrhetinic acids isolated from Glycyrrhiza glabra.^36^ Our finding was in contrast with mentioned studies. Its antioxidant activity was also demonstrated by some researchers^43^^,^^44^ and was noticed to have vasorelaxant effect, anti-platelet, anti-viral and estrogenic activities.^45^ Platelet release was shown to cause an increase in the collagen content resulting into improvement of wound repair.^46^

We showed that 10% licorice extract was not an effective dressing in healing of third degree burns infected to *P. aeruginosa *and purulent exudate was still present. As we administered licorice extract with high concentration (10%), adverse effects of hypokalemia, anti-platelet and corticoid-like activitities may explain its poor healing activities. The presence of purulent exudate of *P. aeroginosa* in our study was previously shown and confirmed in another study.^47^ Licorice with its anti-platelet property can explain to inhibit burn wound tissue healing.^46^

In the present study, licorice gel could not accelerate the burn wound healing in comparison to SSD and wound strength was still poor compared with SSD. One of the most important factors in the healing of burn wounds is the wound strength.^48^ Wound strength is determined by the amount and quality of newly synthesized and deposited collagen, as well as degradation of preformed collagen.^49^ A slow increase in wound tensile strength corresponds to the increase in fibroblasts, which begin to produce immature collagen during the proliferative phase of wound healing.^50^ Fibroblasts play an important role in producing the collagen necessary to restore the tensile strength of wounded skin.^51^

In the latter stage of healing also known as the remodeling phase, the tensile strength is increased as the collagen is reorganized and matured into larger bundles and thus increased the strength of the wound.^52^ Collagen intermolecular cross-links were shown to be essential for providing the stability and tensile strength of the skin. Cross-linking formation took place as the triple helical collagen molecules lined up and began to form fibrils and then fibers. The recovery of tensile strength of healing skin is related to several factors, including healing time and the nutritional status of the healing tissues.^53^ Wound strength happens by remodelling of collagen fibers, and formation of stable intra- and inter-molecular crosslinks.^54^ Even it was shown that 1% and 2% licorice topical gel were effective in reduction of erythema, edema, and itching with anti-inﬂammatory properties, our findings revealed that licorice extract in 10% concentration was not effective in healing of *P. aeruginosa *infected burn wounds and a decrease in inflammation. So this concentration is not considered as a therapeutic dose.

## Footenote

Please cite this paper as:

Tanideh N, Rokhsari P, Mehrabani D, Mohammadi Samani S, Sabet Sarvestani F, Ashraf MJ, Koohi Hosseinabadi O, Shamsian Sh, Ahmadi N. The Healing Effect of Licorice on Pseudomonas aeruginosa Infected Burn Wounds in Experimental Rat Model. World J Plast Surg 2014;3(2):99-106.
